# Pine Extract for Bathing

**Published:** 1884-12

**Authors:** 


**Pine Extract for Bathing.**—It has long been recognized that the atmosphere of pine forests has an invigorating and beneficial effect upon people with weak constitutions and suffering from pulmonary disorders. At some of the watering places of Germany the very simple prescription of the physician is that the patient should spend several hours a day walking or riding through the pine wood. This simple treatment is sometimes supplemented by the taking of pine baths, and in the case of kidney diseases and for delicate children this is claimed to be highly beneficial. The bath is prepared by simply pouring into the water about half a tumblerful of an extract made from the fresh needles of the pine. This extract is dark in color and closely resembles molasses in consistency, and when poured into the bath gives the water a muddy appearance with a slight foam on the surface. The repugnance one feels to enter into such a muddy-looking fluid is dispelled as soon as the delightful aroma which arises from the bath is inhaled. Although there may be some doubt whether pine baths act upon the system in any other way than as a tonic, still, as an adjunct to the daily bath, infusion of the pine extract induces a most agreeable sensation. It gives the skin a deliciously soft and silky feeling, and the effect upon the nerves is quieting. It is a matter of some surprise to us that the business of manufacturing and bottling the extract for private use and public bathing establishments has not been tried in this country, where pine forests abound so extensively. The extract, when properly bottled and securely corked, will not deteriorate for a long time, and the cost for gathering the pine needles and extracting their tarry substance would not be very great, while the demand for it would likely increase to large proportions when the public became accustomed to its use.—*Scientific American.*

At a recent meeting of the Philadelphia County Medical Society, Dr. Carl Seiler called attention to the value of tincture of benzoin in the treatment of chapped hands and frosted feet. He has used it in a number of cases with much success. It is applied by simply painting it on the skin.

